# 

**Published:** 1894-07

**Authors:** 


CONTENTS.

ORIGINAL COMMUNICATIONS—

Suggestion and Its Therapeutic Uses. By Samuel C. Benedict, M.D., Athens, Ga. 257

Amputation and Dressings. By Howard J. Williams, A.M., M.D., Macon, Ga....... 268

The Treatment of Pterygia with the Galvano-Cautery. By Arthur G. Hobbs, M.D., Atlanta, Ga........ 284

Chronic Otorrhea—Its Significance and Treatment. By Dunbar Roy, A.B., M.D., Atlanta, Ga........ 287

SOCIETY REPORTS—

Macon Medical Society........ 296

EDITORIAL—

The Meeting of the American Medical Association........ 299

The Growth of Medical Journalism........ 300

Dr. J. B. S. Holmes........ 302

SELECTIONS AND ABSTRACTS—

Obstetrics and Gynecology........ 303

Skin Diseases and Syphilis........ 307

Practical Chemistry and Pharmacy........ 310

MEDICAL ITEMS........ 313

BOOK REVIEWS........ 317

ADVERTISERS' INDEX TO ADVERTISEMENTS........ xii

CLASSIFIED INDEX TO ADVERTISEMENTS........ xxxvii

ITEMS OF INTEREST........ x, xi, xxxi

